# Impact of Dietary Phytogenic Composite Feed Additives on Immune Response, Antioxidant Status, Methane Production, Growth Performance and Nutrient Utilization of Buffalo (*Bubalus bubalis*) Calves

**DOI:** 10.3390/antiox11020325

**Published:** 2022-02-08

**Authors:** Krishan Kumar, Avijit Dey, Manoj Kumar Rose, Satbir Singh Dahiya

**Affiliations:** 1 Division of Animal Nutrition and Feed Technology, ICAR-Central Institute for Research on Buffaloes, Hisar 125001, Haryana, India; krishen.sheoran36@gmail.com (K.K.); ssdahiyacirb@gmail.com (S.S.D.); 2 Department of Veterinary Physiology and Biochemistry, College of Veterinary Science, Lala Lajpat Rai University of Veterinary and Animal Sciences, Hisar 125004, Haryana, India; manoj.rose@gmail.com

**Keywords:** plant bioactives, eucalyptus leaves, poplar leaves, immune response, antioxidant status, nutrient utilization

## Abstract

The focus on the bioactive effects of plants concerns mainly ruminal microflora for the modulation of rumen fermentation with very little emphasis placed on their consequences on health parameters, including antioxidative and immune stimulating effects. The aim of the present study was to examine the effects of supplementing phytogenic feed additives composed of a mixture of eucalyptus (*Eucalyptus citriodora)* and poplar (*Populus deltoides*) leaf-meal (EPLM) on immune response, antioxidant status, metabolic profile, enteric methane production, growth performance, and nutrient utilization in buffalo (*Bubalus bubalis*) calves. In vitro studies with graded doses of EPLM extract revealed a reduction in total gas and methane production with an increased proportion of propionate without affecting feed degradability. In the in vivo experimentation, eighteen female buffalo calves (10–14 months old, avg. body weight 131.68 ± 7.50 kg) were divided into three groups (CONT, EPLM-1, and EPLM-2) of six each in a completely randomized design. Treatment groups were supplemented with a blend (1:1) of dry grounded eucalyptus (*Eucalyptus citriodora*) and poplar (*Populus deltoids*) leaves (50 g, EPLM-1; 150 g, EPLM-2). Feed intake and growth rate of buffalo calves fed on different feeding regimens did not differ (*p* > 0.05). Haemato-biochemical parameters reveal no variations (*p* > 0.05) among groups, irrespective of period of collection, except the concentration of blood urea, which was decreased (*p* < 0.05) in both treatment groups as compared to the control. The levels of reduced glutathione (GSH), catalase (CAT), and superoxide dismutase (SOD) increased (*p* < 0.05) in EPLM supplemented animals as compared to CONT. The extent of lipid peroxidation (LPO) was reduced (*p* < 0.05) with increased level of EPLM in the diet. The total thiol group (T-SH) was also increased with the supplementation of leaves in the diet. The mean absolute values for skin thickness following the intra-dermal injection of PHA-P were increased (*p* < 0.05) in all the supplemented animals relative to CONT. However, there was no significant difference among the calves fed graded levels of these feed additives in the diet. The Ab titer against *Pasteurella multocida* vaccine was higher (*p* < 0.05) on both day 45 and 90 in both treatments, irrespective of the level of additive supplemented. The enteric methane production was reduced in EPLM supplemented buffaloes; however, digestibility of all the nutrients remained comparable (*p* > 0.05) among the animals. It may be concluded that blends of eucalyptus (*Eucalyptus citriodora*) and poplar (*Populus deltoides*) leaf-meal (50 g/h/d) containing 3.19 g, 2.30 g, and 0.71 g of total phenolics, tannin phenolics, and condensed tannins, respectively, can be used as the phytogenic feed additive for improving antioxidant status and immunity of buffalo calves, and mitigating enteric methane production without affecting performance and nutrient utilization.

## 1. Introduction

The gut microbiota of ruminants is composed of a diverse microbial community of mostly bacteria, protozoa, fungi, and archaea, responsible for feeds digestion for supplying nutrients to the host animals [1]. The symbiotic relationship of the rumen microbes helps the utilization of fibrous feeds and the production of microbial proteins. However, all the microbes present in the rumen are not always good for animals, as some of them might not have any beneficial effect on feed utilization and animal productivity [2]. Protozoa do not have specific role to play in rumen fermentation of animals fed poor quality fibrous feeds [3]. Methanogenic archaea present in the rumen produce methane from the carbon dioxide and hydrogen produced as byproduct of feed fermentation and emitted to the environment via eructation mainly [4]. Livestock production contributes about 37–44% of global methane emissions and is a major source of methane production in the agriculture sector [5]. The excess degradation of dietary proteins by rumen microbes beyond the capacity of microbial protein synthesis results in ammonia emissions, which are, further, converted into nitrous oxide, a potent greenhouse gas triggering environmental pollution [6]. These inefficiencies in the rumen are responsible for a loss of feed energy (5–12%) and nitrogen (25–35%) [7,8], which also attract the attention of animal nutritionists for their minimization and diversion into animal production. Therefore, the modulation of rumen functions to improve animal production and reduce environmental pollution through phytogenic feed additives have gained focus to lessen the use of antibiotics in animal feeds [9] owing to consumer demand for healthy animal products.

In monogastric animals, especially poultry and pigs, phytogenic feed additives or phytobiotics are used as an alternative to antibiotics to increase feed efficiency, nutrient bioavailability, and health status by modulating gut microbiome and host immunity [10]. Many essential oil compounds (cinnamaldehyde, thymol, carvacrol, and eugenol), used either individually or in blends, have been reported to enhance innate immunity and resistance against enteric diseases in poultry, in addition to improving feed efficiency [11]. The effectiveness of a phytochemical depends on its type, composition, active component, inclusion level, and environmental circumstances. The dietary inclusion of phytochemicals in pigs and poultry is reported to decrease intestinal challenges by stabilizing gut microbiota and reducing toxic metabolites produced by pathogenic microbes. A reduction in oxidative stress with increased antioxidant activities in tissues was established in phytochemical supplemented broiler birds. Modulation of the immune system by increased proliferation of immune cells, suppression of proinflammatory cytokines, and enhanced membrane integrity and antibody titer was described in monogastricts with dietary supplementation of phytogenic feed additives [12,13].

Phytochemicals (saponins, tannins, and essential oils) present in feed additives of natural sources are designated to modulate the functioning of the rumen microbiome for reducing methanogenesis and protein degradation with the goal to enhance the nutritional value of feeds and further the abatement of environmental pollution from livestock production [6,14]. The antimicrobial properties of plant bioactive compounds, especially tannins and essential oils, facilitate the modulation of the rumen microbial ecosystem; however, their effects on health performance, including antioxidant status and immune response, is very scanty. Limited investigation into ruminants suggests improvements in antioxidant enzymes and immunity indicators by dietary supplementation of tanniferous leaves or rumen protected capsicum [15,16,17].

Buffaloes are an integral part of the livestock production system in South Asian countries, and 57% of global buffalo population is present in India [18]. Contributing nearly 50% of total milk production, buffaloes play an important role in food and the nutritional security of the country [19]. With the contribution of buffalo meat alone, India is one of the largest beef exporting countries of the world. Poplar (*Populus sp.*) is a deciduous plant widespread in Europe and Asia, and leaves are rich in phytochemicals (phenolic compounds, terpenoids, flavones, flavanones, and many compounds in essential oils) [20,21]. Eucalyptus (*Eucalyptus sp*.) leaves contain an array of volatile essential oil compounds, such as 1,8-cineole, limonene, and α-terpineol, and non-volatile phenolic compounds, such as quercetin, epicatechin, and catechin, which are associated with health benefits [22]. The phytochemicals present in these tree leaves may modulate rumen fermentation towards an inhibition of methanogenesis, as well stimulate the immune system and antioxidant status of animals. However, there is no study investigating the effect of dietary poplar–eucalyptus leaf-meal blends on rumen fermentation, growth performance, and health status in ruminants. Therefore, the present investigation was designed to examine the impact of dietary phytogenic composite feed additives containing a mixture of dried leaves of *P. deltoides* and *E. citriodora* on immune response, antioxidant status, growth performance and nutrient utilization of buffalo (*Bubalus bubalis*) calves. The outcome of the present experiment would explore the potential of these tree leaf blends as a natural feed additive for improving immunity in, and the health status of, buffaloes, in addition to enhancing growth performance and reducing environmental pollution through enteric methane mitigation.

## 2. Material and Methods

The experiment was carried out at the animal nutrition research shed of Animal Farm Section, ICAR-Central Institute for Research on Buffaloes, Hisar, India (29.1203° N, 75.8069° E). Necessary approval (IEAC protocol no. 2/2016) from the Institute Animal Ethics Committee (IAEC) was taken to conduct the study and confirm the care of the animals.

The study was conducted in two phases. An in vitro rumen fermentation study was done to identify the suitable concentrations of poplar (*P. deltoides)* and eucalyptus (*E. citriodora)* leaf-meal blend in modulating rumen fermentation for reducing methane production without affecting feed degradability. The in vivo study was conducted to assess the dietary inclusion of these blends on the production performance and health status of buffalo calves.

### 2.1. In Vitro Fermentation Study

Oat (*Avena sativa* L.) fodder was dried at 60 °C for 48 h in a forced hot air oven and ground in a Wiley mill. The feed sample was passed through a 1 mm sieve before its use as a substrate for the study. The matured leaves of poplar and eucalyptus were collected from the institute campus and dried in a forced hot air oven at 50 °C for 72 h. Each sample of ground leaves was extracted separately with petroleum ether solvent in Soxhlet’s apparatus for 72 h and extracts (150 g/L) were prepared. The final extract was made taking equal proportion of each extract and supplementing at four different concentrations (0, 8.33. 16.67, and 33.33 mL/L of fermentation fluid) to oat hay substrate to obtain four treatments for in vitro study.

#### Fermentation Characteristics, Methane and Volatile Fatty Acids Production

Three rumen-cannulated Murrah buffalo steers (average age, 5 years; body weight, 680 ± 17 kg), fed on a roughage based total mixed ration (roughage: concentrate mixture, 60:40), were used as donors of rumen inoculum. Before offering morning feed, the collected fluid from both solid and liquid phases of rumen was mixed thoroughly and brought to the laboratory in pre-warmed thermos flasks. Rumen liquor was strained through a four-layered muslin cloth in a laboratory under anaerobic conditions with constant flushing of CO_2_, maintained at 39 °C. The fermentation medium was prepared with buffered mineral solution [23] and strained rumen fluid mixed at the ratio of 2:1 under a continuous flow of CO_2_, at 39 °C. The oat hay substrate (200 mg ± 5 mg) was incubated with 30 mL fermentation fluid and various concentrations (0, 8.33, 16.67, and 33.33 mL/L) of leaf-meal extract blends in 100 mL graduated glass syringes (Fortuna^®^, Poulten & Graf Ltd., D 97877 Wertheim, Germany) in triplicates, at 39 °C for 24 h. The syringes were gently shaken manually at every 2 h interval. Three syringes containing only buffered rumen fluid were incubated as blanks. The incubation was terminated at 24 h, and the volume of gas produced was recorded. The net gas production from each treatment was calculated by deducting the gas production in blank syringes.

To estimate methane production, an aliquot of 200 µL gas sample was taken in a graduated Hamilton gas-tight syringe (Hamilton, Hamilton Storage GmbH, Domat/Ems, Switzerland), by puncturing the silicon tube of glass syringes, and injected into a gas chromatograph (NUCON 5700, Nucon Engineers, New Delhi, India) equipped with a stainless-steel column packed with Porapak-Q and a flame ionization detector (FID). An aliquot of 1 mL supernatant from fermented fluid of each syringe was taken in a microcentrifuge tube containing 0.20 mL of 25% metaphosphoric acid, and the mixture was incubated for 2 h at room temperature before centrifugation at 5000× *g* for 10 min to attain a clear supernatant, and stored at −20 °C. The volatile fatty acids (VFA) were analyzed by a gas chromatograph fitted with a chromosorb-101 glass column and a flame ionization detector [24]. The concentration of ammonia-N in the fermentation fluid was determined by the Conway disc method [25]. The syringe contents were transferred individually to 500 mL spoutless beakers by repeated washings with a neutral detergent solution, and refluxed [26] to determine the in vitro true dry matter degradability (IVDMD).

### 2.2. In Vivo Study in Buffalo Calves

Eighteen female Murrah buffalo calves (*Bubalus bubalis*, avg. BW 131.68 ± 7.5 kg, 10–14 months old) were selected from the institute’s herd and randomly divided into three groups of six animals each. All the calves were housed in an open shed with a concrete floor, with a proper arrangement of individual feeding and watering. The nutritional requirement for maintenance and growth was complied with [27] for buffalo calves with a diet of chaffed wheat straw, green oats, and concentrate mixture (20.91% crude protein).

#### 2.2.1. Preparation of Composite Feed Additives and Feeding Experimentation

**Figure 1:**
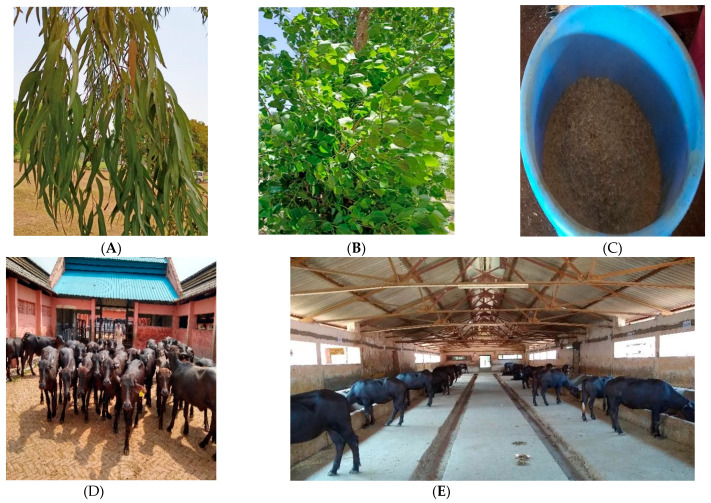
Preparation and supplementation of eucalyptus–poplar leaf-meal blend to buffalo calves. (**A**): Eucalyptus fresh leaves; (**B**): Poplar fresh leaves; (**C**): Blend of eucalyptus–poplar leaf-meal mixed with a small portion of concentrate mixture; (**D**): Experimental buffalo calves; (**E**): The premix was placed in the manger, mixed with the other portion of concentrate mixture, and fed to buffalo calves.

The poplar (*Populus deltoides*) and eucalyptus (*Eucalyptus citriodora*) leaves were harvested from the trees of the institute campus and dried under shade. The leaves were ground in an electric grinder and mixed in equal proportion to make a composite feed additive. The control group animals (CONT) received a control diet (roughage: concentrate, 48:52), while treatment groups received the control diet along with a composite feed additive containing a blend of eucalyptus and poplar leaf-meal (EPLM) at two different dose levels. Each buffalo calf in the treatment groups (EPLM-1 and EPLM-2) were fed EPLM at either 1.3% or 3.9% of feed dry matter intake, respectively, by mixing with a small portion of concentrate mixture (Figure 1) offered daily at 10:30 am. The experimental feeding continued for a period of 100 days, which included 10 days of dietary adaptation and 90 days of actual data recording and measurement. The actual concentration of the additive was achieved on the day 7, with gradual increases from day 1 for proper adaptation to the new feed. Weighed quantities of every feed ingredient (wheat straw, green oats, and concentrate mixture) were provided to establish the precise feed intakes of each animal. Clean drinking water was offered free of choice, three times daily throughout the experimental feeding.

#### 2.2.2. Recording of Body Weight Changes and Feed Efficiency

Weekly body weight changes of each buffalo calves were recorded by taking the body weights of individual animals on two consecutive days in the morning (8:00 am) before offering feed and water by electronic weighing balance, for a period of 90 days. Feed samples collected during the time of feeding and left-over residues on the next day were reserved to determine the feed intake of each buffalo calf. The feed conversion ratio (kg dry matter intake/kg gain) and percentage feed efficiency (kg gain*100/kg dry matter intake) were calculated for all the experimental animals to ascertain the feeding value of the additive on production performance of the buffalo calves.

#### 2.2.3. Immune Response

The immune response on feeding of phytogenic composite feed additive were assessed by examining both cell-mediated immune response (CMI) and humoral immune response (HI) in each buffalo calf [28]. CMI was examined through an in vivo delayed type of hypersensitivity test initiated by injection of phytohaemagglutinin-P (PHA-P). An amount of 150 µg of PHA-P (Sigma-Aldrich Ltd., New Delhi, India, CAS No. 9008-97-3) in 200 μL of phosphate buffer saline solution was injected intra-dermally at two different sites of the neck region. The increased skin thickness due to hypersensitivity reaction was measured by digital Vernier caliper at 0, 24, 48, 72, and 96 h post-injection.

The humoral immune response was examined by measuring the antibody titre after vaccination against haemorrhagic septicaemia (HS). *Pasteurella multocida* culture, inactivated with formaldehyde, in potassium aluminium sulphate adjuvant, was obtained from Haryana Veterinary Vaccine Institute (Department of Animal Husbandry & Dairying, Govt. of Haryana, India). After experimental feeding of 45 days, all buffalo calves were injected subcutaneously with a single dose (5 mL) of vaccine, and blood was collected at 0, 30- and 45-days post-vaccination to study antibody production. About 8 mL of blood was collected from the jugular vein in a heparinized vacutainer, centrifuged at 1800× *g* for 15 min for separation of plasma, and transferred to store at −20 °C. The antibody titer (log_10_) was recorded by measuring optical density for each sample in duplicate at 450 nm by enzyme linked immuno-sorbent assay (ELISA) at the Department of Immunology, Lala Lajpat Rai University of Veterinary and Animal Sciences, Hisar, Haryana, India.

#### 2.2.4. Antioxidant Profile

The antioxidant status of the buffalo calves, as influenced by dietary supplementation of phytogenic composite feed additive, was assessed through the estimation of antioxidant enzymes, viz., reduced glutathione (GSH), catalase (CAT), superoxide dismutase (SOD), lipid peroxidation (LPO), and total thiol (T-SH) groups in the erythrocytes. The blood samples were collected aseptically from the jugular vein of all the calves in 9.5 mL heparinized vacutainers and centrifuged at 1800× *g* for 15 min. The erythrocyte pellet was collected after carefully removing plasma and the buffy coat, and was washed three times with normal saline solution. The erythrocyte pellet (packed RBC) obtained was slowly mixed with an equal volume of chilled distilled water with constant stirring to attain RBC suspension. A working hemolysate of 1:20 dilution was prepared by mixing RBC suspension (0.5 mL) with distilled water (4.5 mL) and was stored at −20 °C until the activities of erythrocytic antioxidant enzymes were estimated.

The erythrocytic GSH was estimated by a dithio-bis-2 nitro benzoic acid (DTNB) method [29] and the CAT activity was measured by the method of Bergmeyer [30].The SOD activity was measured using an MTT (3-[4-5 dimethyl thiazol 2-xl] 2, 5 diphenyl tetrazolium bromide) reduction microtiter plate method and was expressed as mM of MTT formazon formed/mg of hemoglobin [31]. Lipid peroxidation level was assessed by a thiobarbituric acid method [32], in which malonyl dialdehyde (MDA), a product of lipid peroxidation, was estimated. The total thiol (T-SH) group in RBC hemolysate was analyzed following the method of Sedlak and Lindsay [33]. The hemoglobin (Hb) concentration in the hemolysate was measured colorimetrically [34] and the enzyme activities were expressed as per unit of hemoglobin.

#### 2.2.5. Blood Biochemical Parameters

A blood sample (10 mL) was collected in two vacutainers (with or without anticoagulant) from each calf by jugular vein puncture before offering morning feeds and water at 0, 45, and 90 days of the experimental feeding to examine the general wellbeing of the animals. Hemoglobin (Hb) and packed cell volume (PCV) were estimated from whole blood, whereas blood collected without anticoagulant was used to collect sera and stored at −20 °C in deep freeze for biochemical analysis. Alanine amino transferase (ALT), aspartate amino transferase (AST), total protein, albumin, and serum urea concentration were analyzed by commercial biochemical assay kits (Coral Clinical Systems Ltd., Goa, India) through an automated biochemical analyzer (Coralyzer200, Tulip Diagnostics (P) Ltd., Goa, India). The globulin concentration and ratio of albumin to globulin (A:G) were calculated.

#### 2.2.6. Digestibility Trial of Buffalo Calves

A digestibility trial of 8 days duration (2 days adaptation and 6 days collection period) was conducted in the middle of the feeding trial, in which daily feed offered and refusal was recorded to determine DM intake. The daily total fecal output of each buffalo calf was collected in plastic containers, weighed and recorded. After proper mixing of the total feces of each animal, a suitable samp^le^ (1/100th of total feces) was dried at 80 ± 2 °C for 24 h in a forced hot air oven (NSW, New Delhi, India) to determine DM, mixed together animal-wise for 6 day collection, and ground to pass through a 1 mm sieve before being stored for chemical analysis. A porti^on^ (1/400th of total feces) of fresh feces of each calf was collected daily in plastic bottles containing 10 mL 25% (*v*/*v*) sulfuric acid, and was mixed and analyzed for fecal nitrogen content. After analysis of each chemical component in feeds and feces, the nutrient digestibility was calculated for each animal using the average intake and fecal output of each component.

#### 2.2.7. In Vivo Enteric Methane Production Measurement

The eructed gas from each animal was collected at 0 and 90 days of the feeding experiment using a Douglas bag [35]. The exhaled air was collected at 3 h post-feeding for 3 consecutive days, and the concentration of methane was analyzed by gas chromatograph. A Douglas bag consists of an assembly which has a one way valve through which animals can take atmospheric air for breathing, but the exhaled air cannot exit and is directly collected in the bag. One end of the assembly was tightly attached to the mouth of the animal, while the other end of the assembly was attached to the Douglas bag. The exhaled air of buffalo calves was collected and sealed tightly to avoid any leakage, and then analyzed for methane concentration using a gas chromatograph (Nucon 5700, Nucon Engineers, New Delhi, India) fitted with a flame ionization detector (FID) and a stainless-steel column packed with Porapak-Q (length, 1.5; mesh range, 80–100; o.d., 3.2 mm). The oven temperature was 60 °C and the injector and detector temperatures were set to 140 °C and 200 °C, respectively. A 50:50 mixture of methane and carbon dioxide (Centurion Scientific, New Delhi, India) was used as standard. All the samples were analyzed in triplicate along with standards and averages, which were taken for accounts.

#### 2.2.8. Analyses of Feeds, Refusals and Feces

The samples from feeds, refusals, and feces were analyzed as per the Association of Official Analytical Chemists [36] to determine DM by oven drying method (934.01), organic matter by muffle furnace incineration (967.05), crude protein by Kjeldahl method (976.05), ether extract (973.18), and total ash (942.05). Analyses of neutral detergent fiber (NDF) and acid detergent fiber (ADF) were done with sodium sulfite without the use of α-amylase [37]. The extraction of phenolics from eucalyptus and poplar leaves, and the quantification of total phenolics, tannins, and non-tannin phenolics were expressed as tannic acid equivalent [38]. For these, the plant materials were dried at 50 °C using a forced air oven and stored in airtight containers in a cool, dry place after grinding and passing through a 0.5 mm screen. The phenolics were extracted using 70% acetone in water. The total phenolics were estimated using the Folin–Ciocalteu method (Makkar, 2003). Non-tannin phenolics were determined by adding polyvinyl polypyrrolidone (PVPP) to plant extracts to bind tannin phenolics and estimated the phenolics content in the supernatant. The tannin phenolics were calculated by subtracting the non-tannin phenolics from total phenolics (Makkar, 2003). The condensed tannins were quantified by the Butanol-HCl method as leucocyanidin equivalent, as described by Porter, Hrstich, and Chan [39].

#### 2.2.9. Statistical Analysis

The data obtained from the in vitro and in vivo experiments were analyzed for the various parameters as a completely randomized design using a general linear model procedure of SPSS [40] with a fixed effect of treatment. The statistical model, *Y*ij = *μ* + *T*i + *ε*ij was used, where *Y*ij is an individual observation, *μ* is the overall mean, *T*i is the effect of i-th treatment, and *ε*ij is the residual error. The significance of difference between the treatment means was determined by one way ANOVA, as per Snedecor and Cochran [41]. The differences between means were established using Tukey’s HSD test, with significance declared if *p* ≤ 0.05.

## 3. Results

The total gas production after 24 h incubation of oat hay was reduced (*p* < 0.01) in Blend-2 and Blend-3; however, it remained comparable (*p* > 0.05) with the control for Blend-1 (Table 1). The methane concentration (%) in the head space gas and total methane production were reduced (*p* < 0.01) in all the treatments (Blend-1, Blend-2, and Blend-3) as compared to the control. Among the treatments, the methane reduction (*p* < 0.01) was more pronounced in Blend-3 supplementation in comparison to Blend-1 and Blend-2. There was no significant (*p* > 0.05) difference in gas production and methane production between Blend-1 and Blend-2. The concentration of ammonia nitrogen in the fermentation fluid was reduced (*p* < 0.05) in all the treatment groups in comparison to the control. Although differences (*p* < 0.05) were observed among the treatments, the highest reduction was recorded with Blend-3 supplementation, followed by Blend-2 and Blend-1. The inclusion of eucalyptus–poplar leaf-meal extract at any of the experimental dose levels showed comparable (*p* > 0.05) feed degradability (IVDMD) with the non-supplemented control.

Among the volatile fatty acids, acetate production remained similar (*p* > 0.05), irrespective of treatments; however, propionate concentration was increased (*p* < 0.001) in Blend-2 and Blend-3 treatments as compared to the control, while Blend-1 remained comparable with both the control and Blend-2 (Table 1). Butyrate concentration was not affected in either Blend-1 or Blend-2, as compared to the control; however, an increased (*p* < 0.001) concentration was recorded with the supplementation of Blend-3. The increase in propionate production, owing to the supplementation of various blends of eucalyptus–poplar leaves extract, reduced (*p* < 0.01) the ratio of acetate to propionate in all supplemented treatments as compared to the control; however, it was more (*p* < 0.01) pronounced in Blend-3 supplementation. Among the treatments, Blend-1 and Blend-2 were comparable in all these parameters; however, methane production was reduced significantly (*p* < 0.05) in Blend-3 as compared to Blend-1 and Blend-2. Therefore, using equivalent dose levels to treatment groups, Blend-1 and Blend-3 were used as additives for animal feeding in the in vivo experiment.

The basal feed consisted of the concentrate mixture, green oats fodder, and wheat straw (Table 2), confirmed to have sufficient nutrient quality to satisfy the requirements of growing buffalo calves [27]. The protein content of poplar leaves was more than the eucalyptus leaves; however, the crude extracted oil is higher in eucalyptus leaves than poplar, resulting in higher essential oils in the extract. The total phenolics and tannin fractions remained higher in eucalyptus leaves in comparison to poplar leaves, suggesting the presence of higher bioactives. The average initial as well as final body weight of all the experimental buffalo calves (CONT, EPLM-1, and EPLM-2) remained comparable (*p* > 0.05), which ultimately resulted in a similar body weight gain for all the experimental groups. The quantities of concentrate mixture, green oats fodder, and supplements offered to individual buffalo calves during the entire experimental feeding period of 90 days were consumed in whole, resulting in a similar (*p* > 0.05) intake of these feed ingredients. The phytogenic feed supplement provided (per head/day) total phenolics, tannin phenolics, and condensed tannins of 3.19 g, 2.30 g, and 0.71 g and 9.57 g, 6.90 g, and 2.14 g, respectively for EPLM-1 and EPLM-2 group calves. The comparable (*p* > 0.05) total feed intake for all three experimental groups (Table 3), although wheat straw was given ad libitum, culminated in a similar (*p* > 0.05) feed conversion ratio (FCR) as well as feed efficiency (FE).

The skin thickness (%) was highest at 24 h after injection of PHA-P in all the experimental buffalo calves, before subsiding gradually, and coming near to normal after 96 h (Table 4). At any hour post-injection (24, 48, 72, and 96 h), it remained higher (*p* < 0.05) for both groups of EPLM supplemented calves than for the control. However, no difference (*p* > 0.05) was detected between the two supplemented groups (EPLM-1 and EPLM-2), suggesting the triviality of EPLM doses in stimulating the immune system. The antibody titer (log_10_) of buffalo calves supplemented with EPLM was enhanced (*p* < 0.05) at 30 days post-vaccination against *Pasteurella multoicida* compared to the control; however, it remained comparable (*p* > 0.05) between the supplemented calves (EPLM-1 and EPLM-2). A trend of increased (*p* = 0.083) antibody tire was recorded at 45 days post-vaccination in both supplemented groups in comparison to the control (Table 5).

The erythrocytic reduced glutathione (GSH), catalase (CAT), and superoxide dismutase (SOD) activities were enhanced (*p* < 0.01) in both groups of EPLM supplemented buffalo calves in comparison to control. The reduced (*p* < 0.01) lipid peroxidation (LPO) and increased (*p* < 0.01) total thiol groups (T-SH) in both treatment groups (EPLM-1 and EPLM-2), as compared to control group, were evidenced (Table 6). However, no difference (*p* > 0.05) in any of the indicators of antioxidant status was found between the two treatment groups (EPLM-1 and EPLM-2). The hemoglobin (Hb), packed cell volume (PCV), serum protein (albumin, globulin, and A:G ratio), and serum enzyme (ALT and AST) levels did not differ (*p* > 0.05) in experimental buffalo calves, irrespective of feeding regime. However, the serum urea concentration was reduced (*p* < 0.05) in both EPLM supplemented groups in comparison to the control, although no difference (*p* > 0.05) was recorded between the EPLM-1 and EPLM-2 groups (Table 7).

The digestibility coefficients of various constituents (dry matter, organic matter, crude protein, and ether extract) were comparable (*p* > 0.05) in all the experimental calves, irrespective of feeding regime (Table 8). The fiber (neutral detergent fiber and acid detergent fiber) digestibility was not affected (*p* > 0.05) by the dietary supplementation of phytogenic composite feed additives (EPLM-1 and EPLM-2). The daily intake (g/kg W^0.75^) of nutrients, viz., digestible dry matter, digestible organic matter, digestible crude protein, and total digestible nutrients, remained comparable (*p* > 0.05) among the treatments. The nutrient density of the total ration (% digestible crude protein and % total digestible nutrients) was also similar (*p* > 0.05) in all the experimental buffalo calves. The methane concentration (ppm) in the exhaled air before the dosing of the experimental feed additive did not differ (*p* > 0.05) among the calves; however, a trend in the reduction in methane concentration was evident in calves of EPLM-1 (*p* = 0.089) and EPLM-2 (*p* = 0.061) groups at 3 months post-dosing of phytogenic composite feed additive (Table 9). However, the reduction was non-significant (*p* > 0.05), revealing 55.76 and 61.15% decreases in exhaled air methane concentration in EPLM-1 and EPLM-2 groups, respectively.

## 4. Discussion

**Figure 2:**
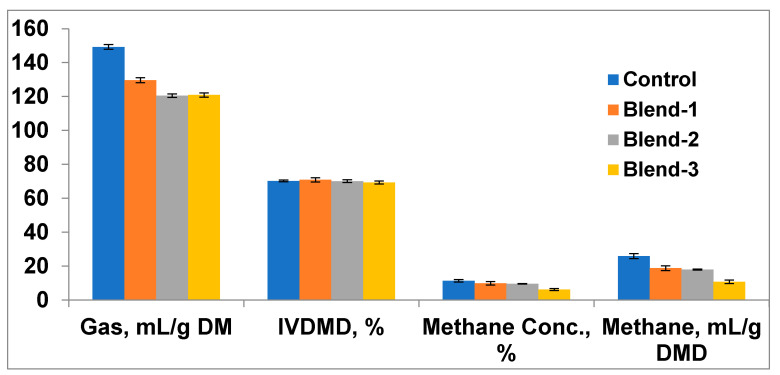
In vitro total gas and methane production as influenced by blends of eucalyptus and poplar leaves extract. DM = Dry matter, DMD = Digestible dry matter, IVDMD = In vitro dry matter degradability.

The dietary modulation of the immune system is described by providing substrates at suitable concentrations to the immune cells, the deprivation of specific nutrients to the invading pathogens, or the direct stimulation of immune cells through hormones and metabolites [42,43]. Phytonutrients can regulate rumen fermentation by altering protein degradation, ruminal ammonia production, nutrient digestion, and volatile fatty acid proportions by modifying the rumen microbiome [6,44]. The reduction in total gas and methane production with supplementation in various blends (Figure 2) in the present study demonstrated specific antimicrobial and antimethanogenic effects of the blends of eucalyptus and poplar leaves extracts, which are rich in phenolics and essential oils [21,22]. The similar in vitro degradability of the substrate in the treatments supplemented with extracts of eucalyptus and poplar leaves with the non-supplemented control signifies that the major fiber degrading microbes were unaffected at the present dose levels. In a study with various phytogenic bioactive compounds on in vitro rumen fermentation, Singh, et al. [45] reported associative effects of plant extracts in modulating rumen fermentation in a dose dependent manner. The reduction in ammonia nitrogen concentration with supplementation of all the blends are suggestive of reduced protein degradation, owing to the inhibition of hyper-ammonia producing bacteria and other microbes involved in amino acid deamination [6,46]. The presence of bioactive compounds in the extract could be associated with the reduced ammonia nitrogen production, as tannins [47,48] and essential oils [49,50] are reported to reduce protein degradation. The shift in volatile fatty acids production with increased concentration of propionate, and thereby the reduced ratio of acetate to propionate, established an alteration of rumen fermentation towards reduced methanogenesis [51,52].

The similar feed intake in all the experimental buffalo calves, irrespective of levels of phytogenic feed additive, is indicative of good palatability of the composite diet. Many researchers [53,54] have reported reduced feed intake owing to the addition of phytogenic compounds in the diet. Similar to the present study, no variation in feed intake was reported when eucalyptus leaf-meal powder was added, either in the diet of beef calves [55] or swamp buffaloes [56]. The reports on dietary supplementation of poplar leaves or composite with eucalyptus leaf-meal on intake and performance of animals are scanty. The feed intake by animals due to inclusion of plant secondary compounds in the diet depends on the type and concentration of bioactive components [49]. Although poplar and eucalyptus leaves are rich in essential oils and phenolics [21,22], a similar feed intake demonstrated no effects on the gut microbiome or digestive enzymes. The comparable nutrient intake and digestibility (Table 8) in all the experimental buffalo calves was advocated by other studies [57,58] with plant bioactive compounds. However, decreased feed digestibility was reported [2] due to a reduction in fiber digestion triggered by the inhibition of ruminal cellulolytic bacteria and anaerobic fungi. The supplementation of eucalyptus leaves demonstrated an increased ADG owing to enhanced nutrient digestibility in buffalo calves [59]; however, reduced feed efficiency and nutrient utilization was reported in other studies with plant compounds [60]. A similar ADG and feed efficiency of buffalo calves to the present study (Table 3) is described by comparable feed intake and digestibility in all the experimental animals.

**Figure 3:**
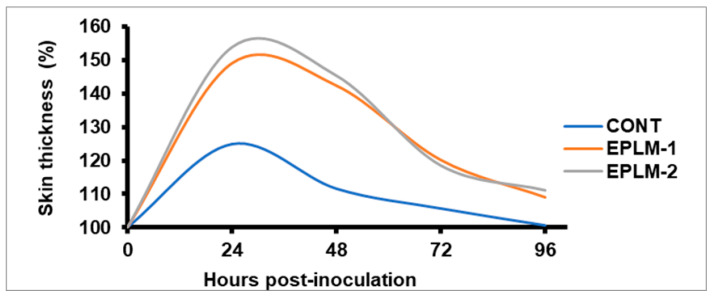
Effect of phytogenic feed additives supplementation on DTH response (%) of buffalo calves to PHA-P. DTH = Delayed type of hypersensitivity; PHA-P = Phytohaemagglutinin-P.

**Figure 4:**
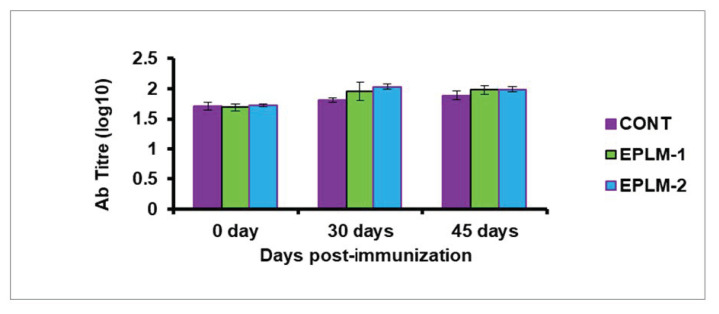
Humoral immune response of buffalo calves measured by antibody titer (log_10_) against *Pasteurella multocida* vaccine.

Although phytogenic bioactive compounds modulate structural and functional properties of gut microflora, the molecular events associated with the mucosa cells and habitat microbes that lead to a modulation of local or systemic immune functions is still unclear [61]. The bioactive plant compounds, viz., tannins, flavonoids, and essential oils, influence the immune systems of animals through different modes of action, such as the binding of proteins to render them unavailable for utilization in rumen and, instead, use quality protein available at lower gut, the interference in active sites of pathogens, or enhancing antioxidant status [62,63]. The increased cell-mediated immune response (Figure 3) and humoral immune response (Figure 4) in all the supplemented calves could be associated with the selective effects of tannins and terpenoids present in the feed additive on gastrointestinal microbes [64]. The probiotic effects of tannins and essential oils through the inhibition of proteolytic and hyper-ammonia producing bacteria [6] with the stimulation of *Bifidobacterium* and *Lactobacillus* [65] could indirectly stimulate the immune system.

The increased levels of indicators (Table 6) of the antioxidant defense system in the supplemented calves are suggestive of a better health status, protecting cells from free radicals through enhanced endogenous antioxidant enzymes [66]. The bioactive compounds present in the EPLM demonstrated enhanced antioxidant indices, as polyphenolic compounds and essential oils [63,67] are reported to enhance these enzymes. The improved antioxidant status also enhanced the immune response of the supplemented buffalo calves. In a study with phytogenic feed additive containing tannins and saponins, Choubey, et al. [68] also reported enhanced antioxidant status and immune response in goats. The absence of any deviation in blood biochemical parameters described the general wellbeing and health of all experimental animals, describing no deleterious effect on liver or other organs owing to phytogenic feed additive supplementation [63,68]; however, a reduced serum urea concentration is indicative of better utilization of dietary protein [69].

The reduction in methane concentration in the exhaled air of calves in supplemented groups advocated an improvement in rumen functions, owing to bioactive compounds (tannins and essential oils) present in the EPLM. The in vitro study with these plant extract blends also confirmed a reduced methane production (Table 1). The inhibition of methane was attributed to a modulation of the rumen microbiome, either through direct inhabitation of methanogens in general or methanogenic archaeal diversity by phenolic compounds and terpenoids present in the supplemented feed additive [6,70]. The present study corroborated the report of Thao, Wanapat, Kang, and Cherdthong [56], in which a reduction in methane production was also demonstrated in swamp buffalo supplemented with eucalyptus leaf-meal.

## 5. Conclusions

The supplementation of phytogenic composite feed additive (EPLM), consisting of an equal proportion of eucalyptus (*Eucalyptus citriodora*) and poplar (*Populus deltoids*) leaves at both the dose levels (50 g and 150 g/h/d) improved the immune response and antioxidant status of buffalo calves with a substantial reduction in methane production. The additive had no detrimental effect on growth and nutrient utilization in buffalo calves. A dose of 50 g/h/d of EPLM, containing 3.19 g, 2.30 g, and 0.71 g of total phenolics, tannin phenolics, and condensed tannins, respectively, is suggested to improve the health status of Murrah buffalo calves without affecting production performance.

## Figures and Tables

**Table 1:** Effects of supplementing blends of the eucalyptus and poplar leaves extract on in vitro total gas production, methanogenesis, ammonia nitrogen, and volatile fatty acids production.

Attributes	Control	Blend-1	Blend-2	Blend-3	SEM	*p* Value
Total Gas, mL	28.33 ^b^	23.67 ^a,b^	23.00 ^a^	22.97 ^a^	0.40	0.001
Gas mL/g DM	149.21 ^b^	124.63 ^a,b^	120.53 ^a^	120.89 ^a^	2.19	0.001
Gas mL/g DMD	212.39 ^b^	175.85 ^a,b^	171.91 ^a^	175.21 ^a^	3.53	0.001
Methane Conc. (%)	11.38 ^c^	9.80 ^b^	9.64 ^b^	6.16 ^a^	0.42	0.009
Total Methane, mL	3.39 ^c^	2.51 ^b^	2.41 ^b^	1.41 ^a^	0.13	0.010
Methane mL/g DM	17.85 ^c^	13.29 ^b^	12.63 ^b^	7.45 ^a^	0.7	0.004
Methane mL/g DMD	25.85 ^c^	18.8 ^b^	18.02 ^b^	10.79 ^a^	0.99	0.009
IVDMD	70.26	70.89	70.11	69.26	0.49	0.447
Ammonia-N, mg/dL	21.93 ^c^	19.6 ^a,b^	20.07 ^b^	18.69 ^a^	0.52	0.046
Acetate, mM/dL	6.56	6.63	6.69	6.83	0.11	0.82
Propionate, mM/dL	1.28 ^a^	1.35 ^a,b^	1.48 ^b^	1.72 ^c^	0.06	<0.001
Butyrate, mM/dL	0.64 ^a^	0.66 ^a^	0.68 ^a^	0.97 ^b^	0.03	<0.001
A:P ratio	5.12 ^c^	4.90 ^b^	4.53 ^b^	3.96 ^a^	0.18	0.003

Control, Blend-1,2,3 are treatment groups, where blend of poplar and eucalyptus leaves extract was supplemented at 0, 8.33, 16.67, and 33.33 mL/L of fermentation fluid, respectively. Mean values bearing a,b,c superscripts in a row vary significantly (*p* < 0.05). DM = Dry matter, DMD = Digestible dry matter, IVDMD = In vitro dry matter degradability.

**Table 2:** Chemical compositions (% DM) of concentrate mixture, wheat straw, green oats, and feed additives consisted of poplar (*Populus deltoides*) and eucalyptus *(Eucalyptus citriodora*) leaves fed to buffalo calves.

Attributes	Concentrate Mixture ^1^	Wheat Straw	Green Oats	Poplar Leaves	Eucalyptus Leaves
*Chemical composition*					
Organic matter	89.17	88.59	89.45	86.54	94.55
Crude protein	20.91	4.64	6.70	11.89	7.33
Ether extract	5.31	0.94	1.80	2.67	7.82
Total ash	10.83	11.41	10.55	13.46	5.45
Neutral detergent fibee	49.63	75.69	68.42	32.75	44.32
Acid detergent fiber	10.83	50.74	43.66	23.46	32.15
*Phenolics fractions*					
Total phenolics ^2^	-	-		4.93	7.82
Total tannin phenolics ^2^	-	-		3.61	5.59
Non-tannin phenolics ^2^	-	-		1.32	2.23
Condensed tannins ^3^	-	-		1.24	1.61

^1^ Concentrate mixture consisted of maize (30%), groundnut cake (15%), mustard cake (20%), wheat bran (32%) mineral mixture (2%) and common salt (1%). ^2^ As tannic acid equivalent. ^3^ As leucocyanidin equivalent.

**Table 3:** Growth rate and feed efficiency of buffalo calves fed phytogenic feed additive blends.

Attributes	Treatments			SEM	*p*-Value
	CONT	EPLM-1	EPLM-2		
**Body weight (kg)**					
Initial	131.77	131.70	131.57	15.38	1.00
Final	180.10	180.52	180.40	20.24	0.936
Total gain ^+^	48.33	48.82	48.83	1.63	0.634
ADG (g)	537.15	542.41	542.59	71.79	0.898
**Dry matter intake (kg/d)**					
Concentrate	1.97	1.99	1.96	0.04	0.820
Wheat straw	1.70	1.69	1.58	0.190	0.816
Green oats	0.14	0.14	0.14	-	-
Supplements *	0.00	0.05	0.15	-	-
Total	3.80	3.86	3.83	0.23	0.960
FCR	7.10	7.2	7.01	0.16	0.815
FE (%)	14.11	14.10	14.14	1.33	0.766

CONT (Control group) = diet containing concentrate mixture, wheat straw, and green oats fodder; EPLM-1 and EPLM-2 = the treatment groups fed the control diet supplemented with a blend (1:1) of eucalyptus and poplar leaf-meal at 50 g and 150 g/hd/day, respectively. ^+^ After 90 days. ADG, Average daily gain; FCR, Feed conversion ratio (kg DMI/kg gain); FE (%), Percent feed efficiency (kg gain*100/kg DM intake). * Supplement consisted of a blend of poplar and eucalyptus leaves powder (1:1).

**Table 4:** Effect of phytogenic feed additive blends on in vivo delayed type of hypersensitivity (DTH) response (%) of buffalo calves to phytohaemagglutinin-P.

Hours Post-Inoculation	Treatments			SEM	*p* Value	Period Mean ± SE
	CONT	EPLM-1	EPLM-2			
0	100	100	100	-	1.00	100 ^A^
24	124.97 ^a^	149.11 ^b^	153.93 ^b^	11.58	0.016	142.67 ^D^ ± 4.72
48	111.59 ^a^	142.45 ^b^	145.44 ^b^	12.10	0.002	133.16 ^C^ ± 4.94
72	105.63 ^a^	120.16 ^b^	118.51 ^b^	6.58	0.042	114.77 ^B^ ± 2.68
96	100.55 ^a^	108.97 ^b^	111.07 ^b^	3.43	0.001	106.86 ^B^ ± 1.40
Treatment mean ± SE	108.55 ^a^ ± 1.86	124.14 ^b^ ± 3.85	125.79 ^b^ ± 4.66	12.29	0.002	

Mean values bearing a, b/A, B, C, D superscripts within a row/column varies significantly (*p* < 0.05).

**Table 5:** Effect of supplementing phytogenic feed additive blends on antibody titer (log_10_) against *Pasteurella multocida* in buffalo calves.

Days Post-Immunization	Antibody Titer (log_10_)			SEM	*p* Value
	CONT	EPLM-1	EPLM-2		
0	1.71	1.69	1.73	0.06	0.244
30	1.81 ^a^	1.95 ^b^	2.03 ^b^	0.07	0.020
45	1.89	1.98	1.99	0.04	0.083

^a,b^ Mean values with different superscript within a row differ significantly (*p* < 0.05).

**Table 6:** Erythrocytic antioxidant indices in buffalo calves supplemented with phytogenic feed additive blends.

Attributes	Treatments			SEM	*p* Value
	CONT	EPLM-1	EPLM-2		
**GSH**					
μmol·mg^−1^ Hb	16.23 ^a^	27.61 ^b^	24.71 ^b^	3.28	0.001
**Catalase**					
mmol·mg^−1^ Hb	0.92 ^a^	7.53 ^b^	5.42 ^b^	0.48	0.006
**SOD**					
mmol MTT formazon formed·mg^−1^ Hb	0.15 ^a^	0.25 ^b^	0.23 ^b^	0.03	0.001
**LPO**					
nmol MDA·mg^−1^ Hb	11.11 ^b^	7.79 ^a^	6.55 ^a^	1.31	0.001
**T-SH**					
μmol·mg^−1^ Hb	209.26 ^a^	360.01 ^b^	309.92 ^b^	42.21	0.001

^a,b^ Mean values with different superscript within a row differ significantly (*p* < 0.01).

**Table 7:** Blood biochemical parameters of buffalo calves supplemented with a diet containing phytogenic feed additive blends.

Parameters	Day	Treatments			SEM	*p* Value
		CONT	EPLM-1	EPLM-2		
Hb (g/dL)	0 day	10.27	11.68	11.38	0.76	0.580
	45 day	11.08	11.73	11.50	0.67	0.647
	90 day	11.13	11.83	11.92	0.71	0.510
PCV (%)	0 day	32.73	34.95	33.60	1.76	0.476
	45 day	32.75	35.67	34.68	1.63	0.198
	90 day	33.10	35.32	34.23	1.61	0.410
AST (U/L)	0 day	120.90	119.27	121.11	5.54	0.572
	45 day	118.32	125.08	123.64	3.23	0.475
	90 day	125.57	118.30	127.16	6.72	0.585
ALT (U/L)	0 day	72.84	76.93	83.51	10.75	0.615
	45 day	69.2	76.63	70.55	6.14	0.462
	90 day	69.53	68.32	69.34	5.76	0.797
Total Protein (g/dL)	0 day	7.63	7.57	7.23	0.23	0.151
	45 day	8.00	8.07	7.67	0.30	0.400
	90 day	7.88	7.90	7.53	0.28	0.367
Albumin(g/dL)	0 day	3.02	3.07	3.02	0.04	0.454
	45 day	3.12	3.13	3.05	0.05	0.307
	90 day	3.07	3.08	3.05	0.068	0.900
Globulin(g/dL)	0 day	4.57	4.48	4.17	0.23	0.203
	45 day	4.88	4.93	4.62	0.27	0.469
	90 day	4.82	4.82	4.48	0.23	0.261
A:G Ratio	0 day	0.68	0.69	0.74	0.04	0.311
	45 day	0.65	0.64	0.66	0.03	0.735
	90 day	0.64	0.64	0.68	0.02	0.143
Urea (mg/dL)	0 day	36.90	39.63	35.65	2.15	0.334
	45 day	41.48 ^b^	34.7 ^a^	37.58 ^a^	3.20	0.017
	90 day	37.85 ^b^	33.10 ^a^	31.93 ^a^	2.20	0.047

^a,b^ Mean values bearing different superscript within a row vary significantly (*p* < 0.05).

**Table 8:** Nutrient digestibility and plane of nutrition of buffalo calves supplemented with phytogenic feed additive blends.

Attributes	Treatments			SEM	*p*-Value
	CONT	EPLM-1	EPLM-2		
**Nutrient digestibility (%)**					
Dry matter	58.93	58.55	59.78	1.90	0.887
Organic matter	61.16	61.39	62.61	1.50	0.888
Crude protein	67.58	66.25	63.79	0.27	0.419
Ether extract	75.16	73.70	75.48	1.96	0.768
Neutral detergent fiber	54.66	55.35	58.50	0.51	0.453
Acid detergent fiber	51.58	48.61	48.68	0.08	0.454
**Nutrient intake (g/kg W^0.75^)**					
Digestible DM	57.72	56.27	54.24	5.48	0.754
Digestible OM	53.98	52.41	50.47	2.80	0.637
Digestible CP	9.25	9.35	8.25	0.56	0.222
TDN	56.68	55.02	52.99	2.94	0.636
**Nutrient Density (%)**					
DCP	13.98	14.68	14.35	0.37	0.348
TDN	57.97	57.28	58.43	1.40	0.825

DCP = Digestible crude protein; TDN = Total digestible nutrients.

**Table 9:** Influence of feeding phytogenic composite feed additives on in vivo methane production (methane concentration in exhaled air, ppm).

Treatments	Methane Conc. (ppm)		SEM	*p* Value
	Pre-Dosing	3 Months Post-Dosing		
**CONT**	**0.214**	0.260	0.09	0.713
EPLM-1	0.220	0.115	0.06	0.089
EPLM-2	0.279	0.101	0.08	0.061

## Data Availability

MDPI Research Data Policies.
